# Validation of the Korean Version of the Positive and Negative Sleep Appraisal Measure (PANSAM) as a Tool for Evaluating Dysfunctional Beliefs about Sleep among the General Population

**DOI:** 10.3390/jcm11164672

**Published:** 2022-08-10

**Authors:** Young Rong Bang, Eulah Cho, Oli Ahmed, Joohee Lee, Lydia Pearson, Junseok Ahn, Seockhoon Chung

**Affiliations:** 1 Department of Psychiatry, Ulsan University Hospital, University of Ulsan College of Medicine, Ulsan 44030, Korea; 2 Department of Psychiatry, Asan Medical Center, University of Ulsan College of Medicine, Seoul 05505, Korea; 3 Department of Psychology, University of Chittagong, Chattogram 4331, Bangladesh; 4 National Centre for Epidemiology and Population Health, Australian National University, Canberra 2601, Australia; 5 School of Health Sciences, University of Manchester, Manchester M13 9PL, UK; 6 Greater Manchester Mental Health NHS Foundation Trust, Manchester M25 3BL, UK

**Keywords:** insomnia, sleep, dysfunctional beliefs, self-assessment, psychology

## Abstract

We explored the reliability and validity of the Korean version of the Positive and Negative Sleep Appraisal Measure (PANSAM) scale using pre-existing sleep-related questionnaires among the general population. Through an online survey, data from 400 South Korean participants were collected from 10 to 18 January 2022. Symptoms were measured with the PANSAM, Insomnia Severity Index (ISI), the 16-item Dysfunctional Beliefs and Attitudes about Sleep Scale (DBAS-16), the Glasgow Sleep Effort Scale (GSES), and the discrepancy between desired time in bed and the desired total sleep time (DBST) index. The four PANSAM subscales were reliable and valid tools for measuring individuals’ dysfunctional beliefs about sleep. A confirmatory factor analysis revealed that the full-scale and four-factor model showed a good fit. The full scale and each subscale were significantly correlated with ISI, DBAS-16, and GSES scores. The DBST index was significantly correlated with Subscales 2 and 3. In conclusion, the Korean version of the PANSAM scale and its four subscales can be applied when clinicians measure dysfunctional beliefs regarding sleep among the general population in South Korea. The PANSAM should be explored among other clinical groups to elucidate its applicability as a trans-diagnostic tool while conducting cognitive behavioral therapy for insomnia.

## 1. Introduction

Almost one-third of the population complains of chronic insomnia. In South Korea, the prevalence of insomnia increased from 3.10% in 2005 to 7.20 in 2013 among women and 1.62% in 2005 to 4.32% in 2013 among men [1]. Persistent or chronic insomnia refers to insomnia occurring at least three times per week for at least three months. In particular, sleep-related maladaptive thoughts may play an important role as one of contributing factors to sleep disturbances [2]. The dysfunctional beliefs in sleep are illogical thoughts of sleep problems, which play an important role in perpetuating or even exacerbating insomnia. For instance, patients with insomnia frequently complain that “sleep problems might interfere with my ability to function during the day” or “as a result of my insomnia, my health will be endangered” [2]. Several studies have examined the association between dysfunctional beliefs about sleep and insomnia symptoms. A meta-analysis of randomized controlled studies has shown that cognitive behavioral therapy for insomnia (CBT-I) has a moderate to large effect in treating insomnia [3]. A study regarding the evaluation of CBT-I effects found that sleep-related dysfunctional beliefs endorsed by patients could be improved by CBT-I [4]. Thus, examining patients’ beliefs about the consequences of insomnia and sleep control could be key to treating chronic insomnia.

There are several questionnaires related to dysfunctional beliefs about sleep. The Dysfunctional Beliefs and Attitudes about Sleep Scale (DBAS) [5] is commonly used for evaluating dysfunctional beliefs, especially for people with insomnia. The DBAS was developed to identify various sleep-disruptive thoughts such as misconceptions about the causes of insomnia, misattribution or amplification of its consequences, and unrealistic sleep expectations. It was derived from clinical experience with patients experiencing insomnia and from psychological conceptualizations of insomnia [6]. Similarly, the Glasgow Sleep Effort Scale (GSES) [7], Insomnia Catastrophizing Scale (ICS) [8], and Anxiety and Preoccupation about Sleep Questionnaire (APSQ) [9] are also used for evaluating dysfunctional cognitions of people with insomnia. However, they do not include maladaptive beliefs that account for the wider range of sleep duration disturbances. Insomnia is included in a complex range of sleep disturbances encompassing sleep duration and variability as well as subjective dissatisfaction with sleep. There is a range of sleep duration disturbances that include not only insomnia but also hypersomnia and reduced need for sleep, which can induce or be induced by a serial change in one’s cognition, behavior, and mood fluctuations.

The Positive and Negative Sleep Appraisal Measure (PANSAM) [10] has been recently developed as a theory-driven measure based on an Integrative Cognitive Sleep Model (ICSM) [11]. The ICSM explains that contradictory appraisals that enter one’s awareness result in engaging in ascent or descent behaviors to control the change of internal state. However, this can lead to a shifted sleep duration or disturbances of the sleep–wake cycle and, thus, psychological distress. If someone feels more energized than usual, for example, they may appraise the need to reduce sleep to take advantage of having more work. This is in contrast to an appraisal of the negative health effects of not sleeping enough. As these appraisals enter awareness, individuals reduce sleep time along with being more productive (ascent behavior), followed by canceling obligations to catch up on sleep (descent behavior). Finally, these behaviors can result in a fluctuating sleep pattern, causing distress about sleep.

According to the PANSAM, positive and negative sleep appraisals for excessively long and short sleep durations play a key role in the development of insomnia, hypersomnia, and reduced need for sleep. Moreover, the PANSAM has shown clinical validity by revealing discrimination between clinical groups of bipolar disorders versus a nonclinical control group [10]. The results suggest that dysfunctional or conflicting beliefs related to sleep can commonly overlap with mood problems, and the PANSAM may be potentially applied as a trans-diagnostic approach from identifying the problems to using CBT for intervention.

The aim of this study was to explore the reliability and validity of the Korean version of the PANSAM scale using pre-existing sleep-related questionnaires among the general population to examine whether the scale can be applied as a tool for measuring the dysfunctional beliefs about sleep.

## 2. Materials and Methods

### 2.1. Participants

This online survey was conducted among the general population in South Korea from 10 to 18 January 2022, via a professional survey company, EMBRAIN (Seoul, Korea) (www.embrain.com). In total, 400 registered panels participated in the survey.

### 2.2. Procedure

Information on participants’ age, sex, living region, marital status, past psychiatric history, and current psychiatric distress was collected. The rating scales included the PANSAM, ISI, DBAS-16, and GSES. The discrepancy between desired time in bed and the desired total sleep time (DBST) Index was also calculated from their responses to questions on the desired time in bed and the desired total sleep time. We developed an online survey form according to the Checklist for Reporting Results of Internet e-Surveys (CHERRIES) guidelines [12], and one investigator (S.C.) tested the usability and technical functionality of the survey form before implementation. The study protocol was approved by the Institutional Review Board of the Asan Medical Center (2021-1755), and the need for obtaining written informed consent was waived.

The sample size was estimated based on the allocation of 40 samples for 10 cells; biological sex (male and female) × five age groups (18–29 years, 30–39 years, 40–49 years, 50–59 years, and 60–80 years) [13]. The sample size required to determine whether a correlation coefficient differs from zero were 396 (type I error (α) = 0.05, type II error (β) = 0.15, and expected correlation coefficient = 0.15). We planned to collect 400 participants’ responses to the survey from the 14 million general population panelists registered in the survey system. An email was sent randomly to 3000 registered panelists, inviting them to participate in the survey. A total of 949 people accessed the survey, and 468 completed it. Four hundred responses were collected after weeding out those with too short or long response times. Data were delivered to investigators after excluding all identifiable personal information.

### 2.3. Measures

We asked participants to respond to each item on all rating scales based on their mental state over the last two weeks.

#### 2.3.1. Positive and Negative Sleep Appraisal Measure

The PANSAM was developed to assess extreme positive and negative sleep appraisals that a person may endorse regarding sleeping more or less than usual. PANSAM development was informed by a Delphi method approach [10]. It consists of 33 items, and an exploratory factor analysis confirmed four theoretically derived subscales [11,14]: Subscale 1 (positive appraisals of sleeping less than usual; Items 8, 12, 15, 16, 20, 24, and 32), Subscale 2 (negative appraisals of sleeping less than usual; Items 2, 6, 10, 14, 18, and 21), Subscale 3 (positive appraisals of sleeping more than usual; Items 3, 7, 11, and 19), and Subscale 4 (negative appraisals of sleeping more than usual; Items 1, 5, 13, 25, and 29). Higher full-scale average scores could be indicative of a person having multiple, conflicting appraisals about sleep, and each subscale’s average scores could be indicative of a person endorsing more positive beliefs about sleeping less (reduced need for sleep) compared to negative beliefs about sleeping less (insomnia).

We translated the PANSAM scale into Korean using translation and back-translation methods. A bilingual expert translated the original English version into Korean. Another bilingual expert translated the Korean version back into English. A third party compared and verified the original English version and the reverse translated English version and found subtle differences between words and expressions. Following this process, we arrived at the final Korean version of the PANSAM scale.

Originally, items could be rated on a visual analog scale from 0 (“I do not believe this at all”) to 100 (“I believe this completely”), and the total score of each subscale was calculated as a mean score of all items in the subscale. In this study, we applied the scale on a Likert-type scale from 0 (“I do not believe this at all”) to 10 (“I believe this completely”), with the same continuous 100 mm VAS in the background and a total score was calculated as the mean score of items in each subscale multiplied by 10. To the best of our knowledge, there are no studies that have explored the comparisons between 0–100 VAS and 0–10 Likert scale of the PANSAM. However, in a validation study of DBAS-16, one of the most popular rating scales for dysfunctional beliefs about sleep, 100 mm VAS was applied with transformation to the 0–10 Likert scale [2]. According to this validation study, we applied the 0 to 10 VAS of the PANSAM scale.

#### 2.3.2. Insomnia Severity Index

The ISI is a self-report rating scale that can measure an individual’s insomnia severity [15]. The total score of seven items of the ISI can range from 0 to 28, and a higher score reflects severe insomnia. The cut-off score of eight was usually applied to define a moderate degree of insomnia. The Korean version of the ISI was already validated [16], and a Cronbach’s alpha of 0.92 and good convergent validity with the Pittsburgh Sleep Quality Index or Epworth Sleepiness Scale were reported. Cronbach’s alpha of the ISI among this sample was 0.80.

#### 2.3.3. Dysfunctional Beliefs and Attitudes about Sleep—16 Items

The DBAS-16 was developed to measure an individual’s dysfunctional beliefs and attitudes about sleep [2]. The 16 items of the DBAS-16 are rated on a Likert-type scale from 0 (strongly disagree) to 10 (strongly agree), and the total score is calculated by adding scores for all 16 items and dividing by 16. A higher total score reflects a higher level of dysfunctional beliefs about sleep. No optimal cut-off score was proposed. The Korean version of the DBAS-16 was validated [17], and Cronbach’s alpha of 0.85 and good fits for the four-factor model (goodness-of-fit-index = 0.88, comparative fit index = 0.85. and root mean square error of approximation (RMSEA) = 0.08) were reported. Cronbach’s alpha among this sample was 0.90.

#### 2.3.4. Glasgow Sleep Effort Scale

The GSES is a self-report rating scale that can measure an individual’s persistent preoccupation with sleep [7]. The seven items of the GSES are rated on a three-point Likert scale from 0 (not at all) to 2 (very much). A higher total score reflects a greater effort to fall asleep. The Korean version of GSES was already validated [18], and a Cronbach’s alpha of 0.76 and test–retest reliability of 0.83 were reported. Good convergent validity was also reported with the ISI and DBAS-16. Cronbach’s alpha for this sample was 0.76.

#### 2.3.5. Discrepancy between Desired Time in Bed and Desired Total Sleep Time: The DBST Index

The DBST index was defined as a difference in one’s desired time in bed from one’s desired total sleep time to measure one’s severity of insomnia [19]. Although it is a new concept used to easily assess an individual’s insomnia severity, we explored the usefulness of the DBST index among the general population. We calculated one’s desired time in bed and total sleep time based on participants’ questions “For what hours do you want to sleep a day?” (desired total sleep time) and “From what time to what time do you want to sleep?” (desired time in bed). The DBST index was calculated as [desired hours of time in bed]–[desired hours of total sleep time].

### 2.4. Statistical Analysis

The psychometric properties of the Korean version of the PANSAM scale were assessed using the classical test theory (CTT) and Rasch analysis. Under the CTT, a confirmatory factor analysis (CFA) was run to test the factor structure of the scale. The CFA model fitness was assessed through the χ^2^/df ratio, comparative fit index (CFI), Tucker–Lewis index (TLI), RMSEA, and standardized root-mean-square residual (SRMR) values [20,21]. Multi-group CFA was conducted to explore whether the four factors model of the PANSAM scale could measure one’s sleep appraisals in the same way across sex and insomnia (ISI score ≥ 8). Furthermore, item analysis was conducted to assess the item–total correlation of items and internal consistency reliability (i.e., Cronbach’s alpha, McDonald’s omega, split-half reliability) of the scale. In the Rasch model, infit mean square (infit MnSQ), outfit MnSQ, item difficulty, item and person separation index, and item and person reliability were estimated. Pearson’s correlation analysis was conducted to examine the convergent validity of the full PANSAM and its subscales with other existing rating scales such as ISI, DBAS-16, GSES, and DBST index. IBM SPSS v26 (IBM Corp., Armonk, NY, USA), JASP v0.14.1 (Eric-Jan Wagenmakers, Amsterdam, The Netherlands), and jMetrik 4.1.1 (J. Patrick Meyer, Charlottesville, VA, USA) were utilized for statistical analysis.

## 3. Results

Among the 400 participants, 51 (12.8%) participants experienced past psychiatric symptoms, and 36 (9.0%) had current psychological distress (Table 1). Participants were residents of Seoul (n = 133, 33.3%), Pusan (n = 21, 5.3%), Daegu (n = 4, 1.0%), Incheon (n = 23, 5.8%), Gwangju (n = 6, 1.5%), Daejeon (n = 10, 2.5%), Ulsan (n = 7, 1.8%), Gyeonggi Province (n = 136, 34.0%), Gangwon Province (n = 4, 1.0%), Chungcheong Province (n = 21, 5.5%), Jeolla Province (n = 13, 3.3%), Gyeongsang Province (n = 11, 2.8%), Jeju Province (n = 9, 2.3%), or Sejong (n = 5, 1.3%).

### 3.1. CFA

Data were suitable, and sampling was adequate for CFA based on the KMO measure (0.945) and Bartlett’s test of sphericity (*p* < 0.001). According to the original paper [11], we conducted the CFA using items for Subscale 1 (Items 8, 12, 15, 16, 20, 24, and 32), Subscale 2 (Items 2, 6, 10, 14, 18, and 21), Subscale 3 (Items 3, 7, 11, and 19), and Subscale 4 (Items 1, 5, 13, 25, and 29). However, we observed the factor loading of Item 29 in Subscale 4 was too low (0.29), and thus, we excluded Item 29 from the final four-factor model (Table 2). The CFA with diagonally weighted least squares showed a good model fit for the Korean version of the PANSAM Korean version scale (χ^2^ = 387.890, df = 203, *p* < 0.001, CFI = 0.986, TLI = 0.984, RMSEA = 0.048, and SRMS = 0.069, Table 3). Although this scale had a good model fit, we observed that the factor loading of Item 29 in Subscale 4 was too low (0.29). Therefore, we excluded Item 29 and conducted a CFA again to test the revised factor structure. It had a better model fit than the previous model (χ^2^ = 311.638, df = 183, *p* < 0.001, CFI = 0.990, TLI = 0.988, RMSEA = 0.042, and SRMS = 0.065).

Factor loadings were observed to range between 0.565 and 0.874 (Table 2). Multi-group CFA results suggested that the four-factor model of the PANSAM can measure individuals’ sleep appraisals in the same way across sex and insomnia.

### 3.2. Rasch Model

Table 4 presents the Rasch model outputs of the PANSAM Korean version scale. Infit and mean squares of all the items were within the recommended range (0.50–1.50), except for Item 14. Furthermore, infit and outfit mean squares were above the recommended range (1.65 and 1.60, respectively). Regarding item difficulty, for Factor 1, Item 15 showed the lowest item difficulty, and Item 8 showed the highest; for Factor 2, Item 14 had the lowest and Item 18 showed the highest item difficulty; for Factors 3 and 4, Items 19 and 1 had the lowest item difficulty, respectively, and Items 11 and 5 had the highest, respectively. All factors indicated an acceptable item separation index, item reliability, person separation index, and person reliability, except Factor 3. That is, the person separation index of Factor 3 was slightly below the recommended cut-off value (2.00).

### 3.3. Reliability and Evidence Based on Relationships to Other Variables

The Korean version of the PANSAM scale showed good reliability of internal consistency. Reliabilities of the full scale (Cronbach’s alpha = 0.936 and McDonald’s Omega = 0.937), Subscale I (Cronbach’s alpha = 0.879 and McDonald’s Omega = 0.8819), Subscale 2 (Cronbach’s alpha = 0.895 and McDonald’s Omega = 0.897), Subscale 3 (Cronbach’s alpha = 0.7919 and McDonald’s Omega = 0.792), and Subscale 4 (Cronbach’s alpha = 0.770 and McDonald’s Omega = 0.772) were good (Table 3). Convergent validity was assessed with the pre-existing rating scales of dysfunctional beliefs about sleep (the DBAS-16 and GSES) and insomnia severity (the ISI and DBST index). The total score of the full scale of PANSAM and its four subscales was significantly correlated with ISI, DBAS-16, and GSES scores (ps < 0.001, Table 5). The DBST index was significantly correlated only with Subscale 2 (r = 0.11, *p* < 0.023) and 3 (r = 0.13, *p* < 0.011).

## 4. Discussion

This study found that the four subscales of the PANSAM demonstrated adequate reliability and validity for measuring individuals’ dysfunctional beliefs regarding sleep. The CFA revealed that the full scale or the four-factor model of the scale showed a good fit for each model. Furthermore, the scores for the total scale and those for each subscale were significantly correlated with the ISI, DBAS-16, and GSES scores. However, the DBST index was significantly correlated with only Subscales 2 (negative appraisals of sleeping less than usual) and 3 (positive appraisals of sleeping more than usual) of the PANSAM.

The Korean version of the full PANSAM scale and its four subscales demonstrated good reliability. Furthermore, the full scale and four subscales demonstrated good validity among the current sample. However, Subscale 4 (negative appraisals of sleeping more than usual) included only four items (Items 1, 5, 13, and 25) since the factor loading value of Item 29, “sleeping too much is a waste of time,” was low. Although the CFA showed a good model fit for the PANSAM scale including Item 29 (χ^2^ = 387.890, df = 203, *p* < 0.001, CFI = 0.986, TLI = 0.984, RMSEA = 0.048, and SRMS = 0.069), we decided to exclude this item from the final model based on its low factor loading value (0.29). Even after excluding Item 29, CFA showed a better fit for the model than previous model (χ^2^ = 311.638, df = 183, *p* < 0.001, CFI = 0.990, TLI = 0.988, RMSEA = 0.042, and SRMS = 0.065). We thus consider Item 29 to be useful for measuring one’s negative appraisal of sleeping. Nevertheless, South Koreans live in a highly competitive society; thus, the nuance of Item 29 does not seem surprising to them, as they are capable of reducing their sleep when working or studying [22]. This might be a reason for the low factor loading value of Item 29.

In the Rasch model, infit and outfit mean squares of Item 14, “If I do not get enough sleep each night, everyone will think I look exhausted” in Subscale 2, are above the recommended range (1.65 and 1.60, respectively). The factor loading value of Item 14 is also slightly low (0.572). In general, items are accepted when their factor loading values are >0.6 [23], but items with values > 0.5 can be acceptable if the scale consistency is good [24]. Since Cronbach’s alpha (0.895) and McDonald’s omega (0.897) of Subscale 2 showed good internal consistency, we adopted Item 14 in the final model.

Significant correlations between the scores of the full scale of PANSAM, its four subscales, and other rating scales such as the ISI, DBAS-16, and GSES showed that the Korean version of the PANSAM scale can be applied to explore individuals’ insomnia characteristics. These results revealed that the PANSAM scale and its four subscales can be used as a tool for assessing individuals’ dysfunctional beliefs about sleep. Subscales 2 and 3 showed higher correlations with ISI, DBAS-16, and GSES than Subscales 1 and 4. This may indicate that the impact of negative appraisals of sleeping less than usual (Subscale 2) and positive appraisals of sleeping more than usual (Subscale 3) are higher than positive appraisals of sleeping less than usual (Subscale 1) and negative appraisals of sleeping more than usual (Subscale 4).

Interestingly, the DBST index was significantly correlated only with Subscales 2 and 3 among this sample. The DBST index reflects the discrepancy between one’s desired time in bed and total sleep time. Insomnia patients often say that “My only wish is to have at least 5~6 h of sleep!” However, they want to stay in their bed for a longer time to have a deep sleep. A higher level of the DBST index might reflect that respondents dysfunctionally want to sleep more, despite their wish to obtain at least a short time of deep sleep. The PANSAM scale was useful for the characterization of these dysfunctional wishes. Subscales 2 and 3 were significantly correlated with the DBST index simultaneously, which may be indicative of the usefulness of the DBST index in exploring an individual’s sleep appraisals.

The PANSAM scale was originally developed to measure positive and negative sleep appraisal for excessively long and short sleep duration [10]. The results showed that the PANSAM scale can be applied as a tool for measuring sleep-related dysfunctional beliefs. In particular, the PANSAM scale has shown to be effective in discriminating between clinical groups with mood disorders versus nonclinical control groups [10]. The results suggest that the PANSAM scale can help explore dysfunctional beliefs about sleep among the general population. However, based on a previous study, the PANSAM may be potentially applied to various mood disorders for identifying sleep-related dysfunctional beliefs while conducting a CBT-I intervention.

We confirmed that the PANSAM scale can be applied to measure dysfunctional beliefs about sleep among the general population with good reliability and validity. The psychometric properties were examined using CTT and modern theory (Rasch measurement theory)—a chief strength of this study. Furthermore, the age and sex ratios were equal.

There are several limitations to this study. First, it was conducted among the general population, not a clinical sample of patients with insomnia. Sleep problems are more diffuse in the general population. Nevertheless, the reliability and validity of a rating scale must be tested across a range of populations [25,26,27]. Validation of the PANSAM scale needs to be performed among clinical samples in future studies. Second, we applied the Likert-type scale rather than a continuous 100 mm visual analog scale (VAS). Participants cannot easily rate their states based on the 100 mm VAS in an online survey. We applied a Likert-type scale similar to the DBAS-16 scale [2]; however, further study is needed to compare the difference in reliability and validity per rating measure (100 mm VAS vs. 11-point Likert-type scale). Third, an anonymous online survey study might lead to selection or self-selection bias. Moreover, participants may misinterpret general questions about mental health, especially if a clinical interview is not conducted in person. Despite this limitation, we decided to conduct an online survey rather than face-to-face interviews to prevent viral transmission during the COVID-19 pandemic. Finally, this study might be limited because we just collected data about participants’ past psychiatric history or their current need for help for current psychiatric symptoms. Future studies may wish to include specific rating scales or face-to-face interviews to measure participants’ anxiety or depression, which might influence their insomnia severity.

## 5. Conclusions

The Korean version of the PANSAM scale and its four subscales can be applied when clinicians formulate dysfunctional beliefs regarding sleep for the general population in South Korea. This scale’s Korean version can help discriminate people’s positive or negative appraisals of sleep more or less than usual. In clinical practice, the PANSAM scale can be applied to explore the appraisals or dysfunctional beliefs of patients who experience sleep problems. Future studies should explore the PANSAM among other clinical groups to elucidate its applicability as a trans-diagnostic tool while conducting CBT-I.

## Figures and Tables

**Table 1:** Clinical characteristics of the study participants (N = 400).

Variable	Mean ± SD, n (%)
**Sex**	
Male, n (%)	204 (51.0%)
Female, n (%)	196 (49.0%)
**Age (Years)**	41.6 ± 10.8
18–29	86 (21.5%)
30–39	90 (22.5%)
40–49	108 (27.0%)
50–59	96 (24.0%)
≥60	20 (5.0%)
**Marital Status**	
Single	186 (46.5%)
Married, with kids	169 (42.3%)
Married, without kids	35 (8.8%)
Others	10 (2.6%)
**Psychiatric History**	
Have you experienced or treated depression, anxiety, or insomnia? (Yes)	51 (12.8%)
Now, do you think you are depressed or anxious, or do you need help for your mood state? (Yes)	36 (9.0%)
**Symptoms Rating**	
Positive and Negative Sleep Appraisal Measure	119.5 ± 50.6
Subscale 1: Positive appraisals of sleeping less than usual	21.2 ± 12.8
Subscale 2: Negative appraisals of sleeping less than usual	25.6 ± 11.4
Subscale 3: Positive appraisals of sleeping more than usual	15.6 ± 8.3
Subscale 4: Negative appraisals of sleeping more than usual	14.0 ± 7.8
Insomnia Severity Index	11.6 ± 5.1
Dysfunctional Beliefs and Attitudes about Sleep-16 items	5.2 ± 1.4
Glasgow Sleep Effort Scale	11.0 ± 2.6
Discrepancy between desired time in bed and desired total sleep	0.8 ± 1.3

SD, standard deviation.

**Table 2:** Item properties of the PANSAM scale among the general population.

Item	M	SD	Skewness	Kurtosis	CITC	CID	Factor Loading
**Subscale 1: Positive Appraisals of Sleeping Less Than Usual**							
**Item 8**	2.443	2.394	0.830	−0.086	0.705	0.857	0.788
**Item 12**	2.630	2.448	0.707	−0.477	0.754	0.850	0.761
**Item 15**	4.018	2.484	0.015	−0.822	0.585	0.872	0.787
**Item 16**	3.528	2.519	0.252	−0.791	0.637	0.866	0.661
**Item 20**	3.150	2.548	0.482	−0.621	0.66	0.863	0.663
**Item 24**	2.930	2.221	0.400	−0.623	0.594	0.870	0.614
**Item 32**	2.465	2.166	0.630	−0.356	0.726	0.855	0.717
**Subscale 2: Negative Appraisals of Sleeping Less Than Usual**							
**Item 2**	3.590	2.356	0.257	−0.666	0.688	0.881	0.830
**Item 6**	4.578	2.281	−0.221	−0.562	0.789	0.866	0.770
**Item 10**	4.275	2.303	−0.110	−0.612	0.768	0.869	0.787
**Item 14**	5.055	2.432	−0.338	−0.553	0.562	0.901	0.572
**Item 18**	3.515	2.347	0.167	−0.746	0.729	0.875	0.874
**Item 21**	4.600	2.363	−0.096	−0.538	0.780	0.867	0.755
**Subscale 3: Positive Appraisals of Sleeping More Than Usual**							
**Item 3**	4.457	2.624	−0.053	−0.816	0.660	0.708	0.674
**Item 7**	3.375	2.408	0.316	−0.565	0.590	0.745	0.757
**Item 11**	2.855	2.638	0.604	−0.664	0.618	0.729	0.759
**Item 19**	4.942	2.894	−0.111	−0.950	0.541	0.773	0.608
**Subscale 4: Negative Appraisals of Sleeping More Than Usual**							
**Item 1**	3.793	2.522	0.096	−0.948	0.519	0.743	0.719
**Item 5**	3.317	2.4519	0.352	−0.634	0.571	0.716	0.565
**Item 13**	3.420	2.5524	0.261	−0.946	0.624	0.687	0.783
**Item 25**	3.438	2.6274	0.380	−0.740	0.574	0.715	0.620

**Table 3:** Scale-level psychometric properties of the Korean version of the PANSAM scale.

Psychometric Property	Score					Suggested Cut-Off
	F1	F2	F3	F4	Full	
**Cronbach’s alpha**	0.879	0.895	0.791	0.770	0.936	≥0.7
**McDonald’s Omega**	0.881	0.897	0.792	0.772	0.937	≥0.7
**Split-half reliability**	0.872	0.876	0.833	0.785	0.932	≥0.7
**Standard error of measurement**	4.450	3.700	3.790	3.746	12.796	Smaller than SD/2
**Model Fits of Confirmatory Factor Analysis**						
**Model**	**Four-Factor Model**		**Full Scale**		**Suggested Cut-Off**	
**χ^2^ (df, *p*-value)**	387.890 (203, <0.001)		311.638 (183, <0.001)		Non-significant	
**CFI**	0.986		0.990		>0.95	
**TLI**	0.984		0.988		>0.95	
**RMSEA**	0.048		0.042		<0.08	
**SRMR**	0.069		0.065		<0.08	

**Table 4:** Item fits and difficulties of the Korean version of the PANSAM scale through the Rasch model.

Item	Infit MnSq	Outfit MnSq	Difficulty	Item		Person	
				Separation Index	Reliability	Separation Index	Reliability
**Subscale 1: Positive Appraisals of Sleeping Less Than Usual**							
**Item 8**	0.94	0.92	0.21	5.824	0.971	2.425	0.854
**Item 12**	0.82	0.78	0.14				
**Item 15**	1.19	1.21	−0.34				
**Item 16**	1.11	1.15	−0.18				
**Item 20**	1.10	1.09	−0.05				
**Item 24**	1.04	1.25	0.03				
**Item 32**	0.74	0.70	0.20				
**Subscale 2: Negative Appraisals of Sleeping Less Than Usual**							
**Item 2**	1.08	1.06	0.30	6.634	0.978	3.055	0.903
**Item 6**	0.72	0.72	−0.13				
**Item 10**	0.80	0.81	0.00				
**Item 14**	1.65	1.60	−0.35				
**Item 18**	0.95	0.97	0.33				
**Item 21**	0.81	0.85	−0.14				
**Subscale 3: Positive Appraisals of Sleeping More Than Usual**							
**Item 3**	0.82	0.81	−0.15	7.518	0.983	1.981	0.797
**Item 7**	0.93	0.93	0.14				
**Item 11**	0.96	0.94	0.30				
**Item 19**	1.26	1.20	−0.29				
**Subscale 4: Negative Appraisals of Sleeping More Than Usual**							
**Item 1**	1.10	1.09	−0.11	1.960	0.794	1.807	0.766
**Item 5**	0.96	0.97	0.07				
**Item 13**	0.89	0.90	0.03				
**Item 25**	1.04	1.05	0.02				

**Table 5:** Correlation coefficients of each variable in all participants.

Variable	Age	1	2	3	4	5	6	7	8
**1. PANSAM total score**	−0.06								
**2. PANSAM subscale 1**	−0.02	0.89 **							
**3. PANSAM subscale 2**	−0.08	0.82 **	0.58 **						
**4. PANSAM subscale 3**	−0.03	0.84 **	0.68 **	0.71**					
**5. PANSAM subscale 4**	−0.12 *	0.84 **	0.72 **	0.59 **	0.67 **				
**6. ISI**	−0.02	0.42 **	0.29 **	0.43 **	0.43 **	0.34 **			
**7. DBAS-16**	−0.07	0.63 **	0.42 **	0.74 **	0.61 **	0.45 **	0.54 **		
**8. GSES**	−0.09	0.61 **	0.49 **	0.56 **	0.56 **	0.47 **	0.57 **	0.56 **	
**9. DBST index**	−0.08	0.09	0.06	0.11 *	0.13 *	0.02	0.20 **	0.17 **	0.15 **

PANSAM, Positive and Negative Sleep Appraisal Measure; ISI, Insomnia Severity Index; DBAS-16, Dysfunctional Beliefs and Attitudes about Sleep-16 items; GSES, Glasgow Sleep Effort Scale; DBST, Discrepancy between desired time in bed and desired total sleep time. Subscale 1: Positive appraisals of sleeping less than usual. Subscale 2: Negative appraisals of sleeping less than usual. Subscale 3: Positive appraisals of sleeping more than usual. Subscale 4: Negative appraisals of sleeping more than usual. * *p* < 0.05, ** *p* < 0.01.

## Data Availability

Not applicable.
